# Movement smoothness and turning deficits during the timed up and go in older adults with chronic neck pain

**DOI:** 10.3389/fspor.2026.1909320

**Published:** 2026-08-27

**Authors:** Thanya Madsalae, Tanapat Thongprong, Nithinun Chaikeeree, Rumpa Boonsinsukh

**Affiliations:** Department of Physical Therapy, Faculty of Physical Therapy, Srinakharinwirot University, Nakhonnayok, Thailand

**Keywords:** fall, gait, geriatrics, accelerometer, non-communicable diseases

## Abstract

**Background:**

This study investigated phase-specific performance, movement smoothness, and turning kinematics during the Timed Up and Go (TUG) test in older adults with and without chronic neck pain (CNP).

**Methods:**

Eighty-five older adults [CNP, *n* = 25; control (CON), *n* = 60] completed an instrumented TUG (iTUG) using wearable inertial sensors. Total TUG duration, spatiotemporal gait parameters (gait velocity, stride length, and cadence), and phase durations (sit-to-stand, walking forward, turning, walking back, and turn-to-sit) were extracted. Trunk movement smoothness was quantified using the Log Dimensionless Jerk based on acceleration (LDLJa), and peak trunk angular velocities during turning and turn-to-sit were calculated.

**Results:**

Compared with the CON group, participants with CNP required significantly longer to complete the TUG, particularly during the turning, walking-back, and turn-to-sit phases, despite similar gait velocity, stride length, and cadence. The CNP group also demonstrated lower LDLJa values, indicating reduced movement smoothness, together with lower peak trunk angular velocities during turning and turn-to-sit. LDLJa was positively correlated with turning angular velocity in both groups, indicating that smoother movement was associated with faster, more coordinated turning.

**Conclusion:**

Older adults with CNP exhibited impaired functional mobility characterized by prolonged TUG performance and reduced movement smoothness despite preserved basic spatiotemporal gait parameters. These impairments were most evident during dynamic postural transitions, suggesting that phase-specific iTUG metrics and LDLJa provide complementary information on movement quality beyond conventional TUG outcomes and may enhance the clinical assessment of mobility impairment in older adults with CNP.

## 1 Introduction

Chronic neck pain (CNP) is a prevalent musculoskeletal condition among older adults and is frequently associated with substantial disability and reduced quality of life (1, 2). These impairments are often accompanied by gait disturbances, including slower walking speed, shorter stride length, and greater gait variability, which are thought to represent compensatory strategies for maintaining stability (3–5). Conventional gait assessments primarily quantify spatiotemporal parameters such as gait velocity, stride length, and cadence. Although these measures provide valuable information, they may overlook subtle impairments in neuromotor control that precede overt gait abnormalities. Consequently, movement smoothness, which reflects the central nervous system's ability to generate continuous and coordinated motor commands, has emerged as a sensitive indicator of motor control efficiency (6).

Unlike straight-line walking, turning is a biomechanically demanding task that requires precise coordination of body segments, continuous realignment of the body's center of mass over a changing base of support, and rapid adaptation of the gait pattern (7–9). These increased sensorimotor demands make turning particularly sensitive to subtle impairments in motor control that may not be detected during conventional straight-line gait assessment. Consequently, evaluating dynamic postural transitions, such as the turning and turn-to-sit phases of the Timed Up and Go (TUG) test, may provide greater insight into mobility impairment than conventional spatiotemporal gait measures alone.

Movement quality can be quantified using the Log Dimensionless Jerk based on acceleration (LDLJa), which complements conventional gait measures by providing a dimensionless index of movement smoothness. LDLJa normalizes the integrated squared jerk of the acceleration signal with respect to movement duration and amplitude, thereby reducing the influence of these factors and facilitating comparisons of movement smoothness across tasks with different durations and amplitudes (10, 11). Higher (less negative) LDLJa values indicate smoother, more coordinated trunk motion, whereas lower values reflect more irregular and segmented movement consistent with impaired sensorimotor control. Although jerk-based metrics and instrumented TUG (iTUG) analyses have been widely validated for assessing mobility and balance impairments in older adults and other clinical populations (12–14), their application in individuals with CNP remains limited. To our knowledge, no study has combined LDLJa with phase-specific iTUG analysis to evaluate movement quality during demanding postural transitions, particularly turning and turn-to-sit, in older adults with CNP.

This study aimed to examine gait performance and movement quality during the TUG test in older adults with and without CNP. We hypothesized that, compared with older adults without CNP (CON), those with CNP would demonstrate prolonged TUG durations, lower peak trunk angular velocities during turning, and reduced movement smoothness (lower LDLJa values), reflecting impaired sensorimotor control. We further hypothesized that turning velocity would be positively associated with movement smoothness in the CNP group.

## 2 Materials and methods

### 2.1 Participants

Sample size was estimated based on the study by Quek et al. (5), which reported significantly longer total TUG durations in older adults with chronic neck pain (CNP) than in healthy controls. Assuming a two-sided significance level of 0.05, a statistical power of 80%, and an effect size of 0.95 derived from total TUG duration, a minimum of 20 participants per group was required. To improve the precision and robustness of the analyses, participant recruitment was extended beyond the minimum sample size, resulting in the enrollment of 25 older adults with CNP and 60 healthy older adults [control (CON) group], all aged 60 years or older.

All participants were required to ambulate independently without the use of walking aids. Participants in the CNP group had to report neck pain and stiffness, with or without radiating pain, persisting for at least three months and a minimum pain intensity of 3 cm on a 10-cm Visual Analog Scale (VAS). Participants in the CON group were required to have no current or previous history of CNP, no complaints of dizziness or vestibular symptoms, and no musculoskeletal pain that interfered with walking or activities of daily living. Exclusion criteria for both groups included conditions that could influence gait or postural performance, including a history of head or neck trauma; orthopedic surgery or fractures within the previous six months; acute musculoskeletal injury; inflammatory joint disease or arthritis requiring active treatment; systemic disease; known or suspected vestibular disorders or vertigo; neurological disorders; use of medications known to affect balance; and cognitive impairment, defined as a Montreal Cognitive Assessment (MoCA) score <24/30.

The study was approved by the Human Research Ethics Committee of Srinakharinwirot University (SWUEC-039/2562F), and all participants provided written informed consent before participation.

### 2.2 Measurement tools

Demographic characteristics, including age, sex, and body mass index (BMI), were obtained through participant interviews and medical records. All participants also completed questionnaires regarding medication use and comorbidities. Neck pain intensity was assessed using a 10-cm Visual Analog Scale (VAS), representing the average pain experienced during the previous week. Neck-related disability was evaluated using the Thai version of the Neck Disability Index (NDI) (15), administered as an interviewer-assisted questionnaire (16). Balance confidence was assessed using the Thai version of the Activities-specific Balance Confidence (ABC) Scale, with higher scores indicating greater balance confidence. Self-perceived disability associated with dizziness was evaluated using the Dizziness Handicap Index (DHI), in which higher scores indicate greater perceived handicap (maximum score = 100) (17).

The Timed Up and Go (TUG) test was conducted in a controlled laboratory by two researchers. One investigator provided standardized instructions and demonstrations, whereas a second investigator accompanied participants throughout testing to ensure safety. The investigator responsible for processing the sensor data was blinded to group allocation. The instrumented TUG (iTUG) was performed using the APDM Mobility Lab system (APDM Inc., Portland, OR, USA) (18, 19), which consists of wearable inertial sensors and dedicated software for quantitative analysis of TUG performance. Six three-dimensional inertial sensors were attached to standardized anatomical landmarks: the sternum (2 cm below the sternal notch), the fifth lumbar vertebra (near the body's center of mass), the dorsum of both hands, and both shanks (4 cm above the ankle joints). Each sensor incorporated a triaxial accelerometer and gyroscope and recorded acceleration and angular velocity at a sampling frequency of 200 Hz. Sensors positioned on the sternum and wrists were included to maintain the standard full-body APDM configuration but were not analyzed in the present study. Data from the shank sensors were used to derive spatiotemporal gait parameters (gait velocity, stride length, and cadence), whereas data from the lumbar sensor were used for phase segmentation and calculation of the Log Dimensionless Jerk based on acceleration (LDLJa).

Participants performed the TUG barefoot to minimize the influence of footwear on gait kinematics and plantar somatosensory input. To ensure consistency across participants, the turning direction was standardized to the left regardless of the side of neck pain. After one practice trial, participants completed three recorded trials separated by one-minute rest intervals to minimize fatigue. Participants began the test seated on a standard armless chair (seat height: 43 cm) and were instructed to stand up without using their hands, walk 3 m at a comfortable self-selected pace, turn 180° around a marker cone positioned 3 m from the front legs of the chair, return to the chair, and sit down. The average of the three recorded trials was used for analysis. Total TUG duration was derived exclusively from the inertial sensor data rather than stopwatch timing and was calculated continuously from the onset of the sit-to-stand movement to completion of the turn-to-sit phase. Accordingly, total TUG duration represented the sum of the five constituent phases: sit-to-stand, walking forward, turning, walking back, and turn-to-sit.

### 2.3 Data processing and phase segmentation

Spatiotemporal gait parameters were computed automatically using the APDM Mobility Lab software and exported as processed variables. Raw acceleration and angular velocity signals from the lumbar sensor were exported separately for offline analysis using custom scripts written in MATLAB (The MathWorks Inc., Natick, MA, USA). Before phase segmentation and smoothness analysis, the lumbar sensor signals were low-pass filtered using a zero-lag fourth-order Butterworth filter with a cut-off frequency of 10 Hz to remove high-frequency noise.

The five phases of the TUG test (sit-to-stand, walking forward, turning, walking back, and turn-to-sit) were identified from the filtered lumbar sensor signals positioned over the fifth lumbar vertebra, which approximates the body's center of mass. The primary peak in the pitch-axis angular velocity (sagittal plane) was used to identify the sit-to-stand transition, whereas prominent peaks in the yaw-axis angular velocity (transverse plane) were used to identify the turning and turn-to-sit phases. The onset and offset of each phase were defined as the points at which angular velocity exceeded and subsequently fell below 10% of the peak angular velocity for the corresponding movement.

Initial phase segmentation was performed semi-automatically and subsequently verified by visual inspection. When necessary, phase boundaries were manually adjusted by a single investigator who remained blinded to participant group allocation throughout data processing. To evaluate the consistency of phase segmentation, 10 recordings (five CNP and five CON) were randomly selected and independently reanalyzed by the same investigator after a two-week interval. Intra-rater reliability was assessed using a two-way mixed-effects model with absolute agreement [ICC (3,1)]. Excellent reliability was observed for the onset and offset of all TUG phases (ICC = 0.82–0.93), with mean absolute differences between repeated segmentations of less than 0.10 s (0.05 ± 0.02 s).

### 2.4 Movement smoothness (LDLJa)

Movement smoothness was quantified using the Log Dimensionless Jerk based on acceleration (LDLJa), calculated over the entire TUG duration according to previously described methods (11, 20). LDLJa was calculated as:

$$\mathrm{LDL}J_{a}=-\ln\left(\frac{t_{2}-t_{1}}{a_{\mathrm{peak}}^{2}}\overset{t_{2}}{\underset{t_{1}}{\int }}\;{\left|\frac{d}{\mathrm{dt}}a(t)\right|}^{2}\mathrm{dt}\right)$$

where a(t) is the resultant (Euclidean norm) of the three trunk acceleration components, t_₁_ and t_₂_ correspond to the onset of the sit-to-stand transition and the completion of the turn-to-sit, respectively; and a_peak_ is the peak resultant acceleration over that interval. The negative sign was included so that higher (less negative) LDLJa values represent smoother and more coordinated movement.

### 2.5 Statistical analysis

All statistical analyses were performed using SPSS version 31 (IBM Corp., Armonk, NY, USA). Descriptive statistics were calculated to summarize participant characteristics, with continuous variables presented as mean ± standard deviation (SD) or median [interquartile range (IQR)], as appropriate, and categorical variables as frequencies and percentages.

All statistical tests were two-sided. Data normality was assessed using the Shapiro–Wilk test. Gait velocity, stride length, cadence, phase durations, and peak trunk angular velocities were normally distributed and were compared between groups using independent-samples *t*-tests. Homogeneity of variance was assessed using Levene's test for each variable. When the assumption of equal variances was violated, Welch's *t*-test was applied, and the adjusted degrees of freedom are reported. Because variability in phase duration was itself of interest, Levene's *F* statistic together with the group standard deviations are reported for each TUG phase. All between-group differences and effect sizes were calculated as CON minus CNP; therefore, negative values indicate higher values in the CNP group, whereas positive values indicate higher values in the CON group.

Total TUG duration and LDLJa were not normally distributed and were therefore compared using the Mann–Whitney *U* test. Between-group differences for these variables are presented as the Hodges–Lehmann median difference with corresponding 95% confidence intervals (CIs). Because the Hodges–Lehmann estimator represents the median of all possible pairwise between-group differences, it does not necessarily equal the arithmetic difference between the two group medians. The relationships between LDLJa and peak trunk angular velocity during the turning and turn-to-sit phases were examined using Spearman's rank correlation coefficient (*r*_s_). Correlation strength was interpreted according to Cohen's criteria: 0.10–0.29 (small), 0.30–0.49 (moderate), and ≥0.50 (large) (21).

Statistical significance was set at *p* < 0.05. Effect sizes were reported as Cohen's *d* for independent-samples *t*-tests and as *r* = |*z*|/√*N* for Mann–Whitney *U* tests. Ninety-five percent confidence intervals were reported for all between-group comparisons. Because each outcome variable involved a single pre-specified comparison between two independent groups, no adjustment for multiple comparisons was applied.

## 3 Results

The demographic and clinical characteristics of the participants are summarized in Table 1. As expected, the CON and CNP groups were comparable in age, sex, BMI, comorbidities, and medication use. Participants with CNP reported significantly greater neck-related disability, pain intensity, reduced balance confidence, and greater dizziness-related disability than the CON group.

**Table 1:** Characteristics of participants.

Variable	CON (*n* = 60)	CNP (*n* = 25)
Age (years, mean ± SD)	64.70 ± 3.74	64.04 ± 3.75
Gender [female, *n* (%)]	46 (76.67)	18 (72.00)
BMI (kg/m^2^, mean ± SD)	23.56 ± 2.97	23.68 ± 3.87
NDI (%, mean ± SD)	0.63 ± 1.35	15.28 ± 6.22*
ABC scale (%, mean ± SD)	94.08 ± 5.79	89.17 ± 10.17*
DHI [points, median (IQR)]	0.00 (0.00 to 0.00)	0.00 (0.00 to 2.00)*
VAS (0–10, mean ± SD)	–	4.64 ± 1.50*
Duration of neck pain (months, mean ± SD)	–	14.14 ± 13.82
Side of neck pain [sides, *n* (%)]		
Right side	–	6 (24.00)
Left side	–	4 (16.00)
Both sides	–	15 (60.00)
Comorbidities [conditions, median (IQR)]	1.00 (1.00 to 1.00)	1.00 (1.00 to 1.00)
Taking more than four medications [*n* (%)]	5 (8)	3 (12)

CON, older adults without chronic neck pain; CNP, older adults with chronic neck pain; NDI, neck disability index; ABC, activities-specific balance confidence scale; DHI, dizziness handicap index; VAS: Visual Analog Scale.

* *p* < 0.05 indicates a statistically significant difference between the CON and CNP groups.

The gait outcomes are summarized in Table 2. Total TUG duration was significantly longer in the CNP group than in the CON group (Hodges–Lehmann median difference = −1.59 s, 95% CI: −2.29 to −0.93; *p* < 0.001). In contrast, gait velocity, stride length, and cadence did not differ significantly between groups.

**Table 2:** Comparison of gait parameters between older adults with and without chronic neck pain.

Variable	CON (*n* = 60)	CNP (*n* = 25)	Difference (95% CI)	*p*-value	Effect size
Total Duration (s)^†^	11.35 (10.08 to 12.03)	12.84 (12.10 to 13.36)	−1.59 (−2.29 to −0.93)	<0.001*	0.47
Gait Velocity (m/s)^‡^	1.12 ± 0.16	1.06 ± 0.15	0.06 (−0.02 to 0.13)	0.123	0.37
Stride Length (cm)^‡^	108.00 ± 14.09	105.70 ± 13.48	2.30 (−4.29 to 8.90)	0.489	0.17
Cadence (steps/min)^‡^	121.55 ± 14.14	118.08 ± 10.01	3.46 (−2.73 to 9.66)	0.269	0.27

CON, older adults without chronic neck pain; CNP, older adults with chronic neck Pain.

* *p* < 0.05 indicates a statistically significant difference between the CON and CNP groups.

† Variable analyzed using the Mann–Whitney U test due to non-normal distribution. Data are presented as Median (Interquartile Range). The difference is expressed as the Hodges–Lehmann median difference and its 95% CI.

‡ Variables analyzed using the Independent Samples t-test. Data are presented as Mean ± Standard Deviation (SD). The difference is expressed as the mean difference and its 95% CI.

**Figure 1:**
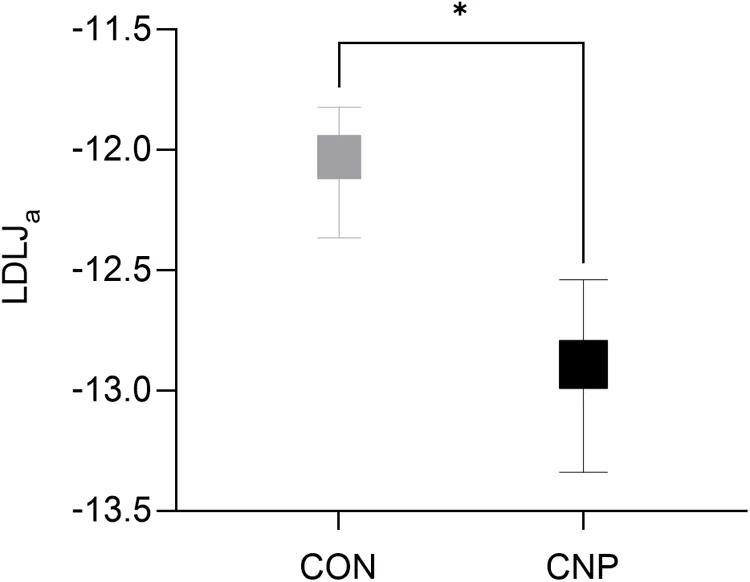
Log dimensionless jerk based on acceleration. Median and interquartile range (IQR) plots comparing the log dimensionless jerk based on acceleration (LDLJa) during the TUG test between the CON and CNP groups. The central squares represent the median values, and the error bars indicate the IQR. Higher (less negative) values indicate smoother movement, whereas lower (more negative) values indicate less smooth movement.

Movement smoothness, quantified using LDLJa, also differed significantly between groups (Figure 1). Participants with CNP demonstrated lower LDLJa values, indicating less smooth trunk movement during the TUG, than the CON group (median difference = 0.79, 95% CI: 0.58–1.01; *p* < 0.001).

**Figure 2:**
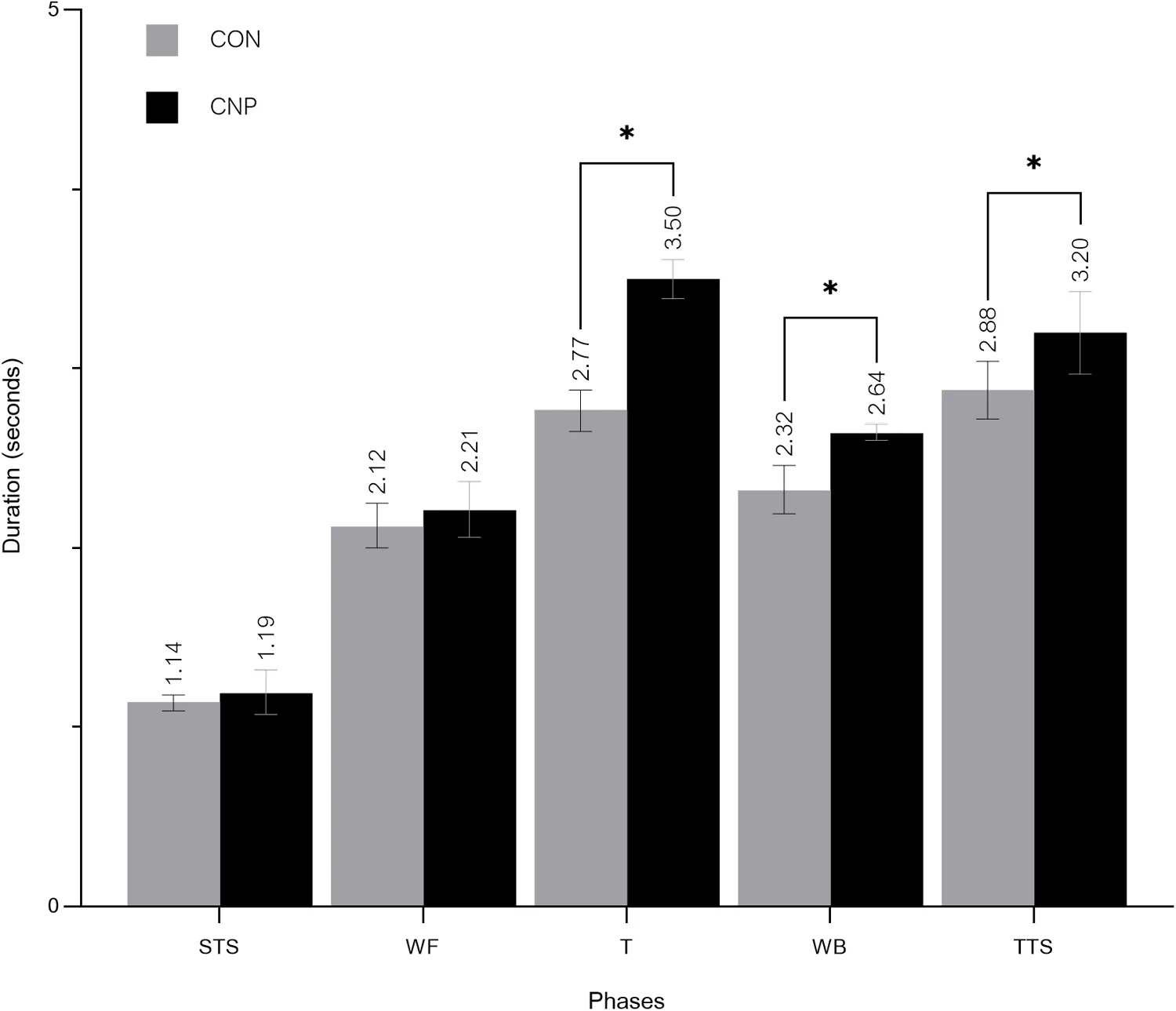
TUG phase duration in older adults with and without CNP. This bar chart compares the duration of TUG phases (seconds) between the CON and CNP groups. The individual phases include Sit-to-Stand (STS), Walking Forward (WF), Turning (T), Walking Back (WB), and Turn-to-Sit (TTS). Significant differences (*p* < 0.05) between groups are marked with an asterisk (*).

Phase-specific TUG durations are presented in Figure 2. Compared with the CON group, participants with CNP required significantly longer to complete the turning, walking-back, and turn-to-sit phases, whereas no between-group differences were observed for sit-to-stand or walking-forward duration. Variability in phase duration also differed between groups. Turning and walking-back durations were significantly less variable in the CNP group, whereas sit-to-stand duration was more variable. No between-group differences in variability were observed for walking forward or turn-to-sit. Accordingly, Welch's *t*-test was applied to comparisons in which homogeneity of variance was violated.

**Figure 3:**
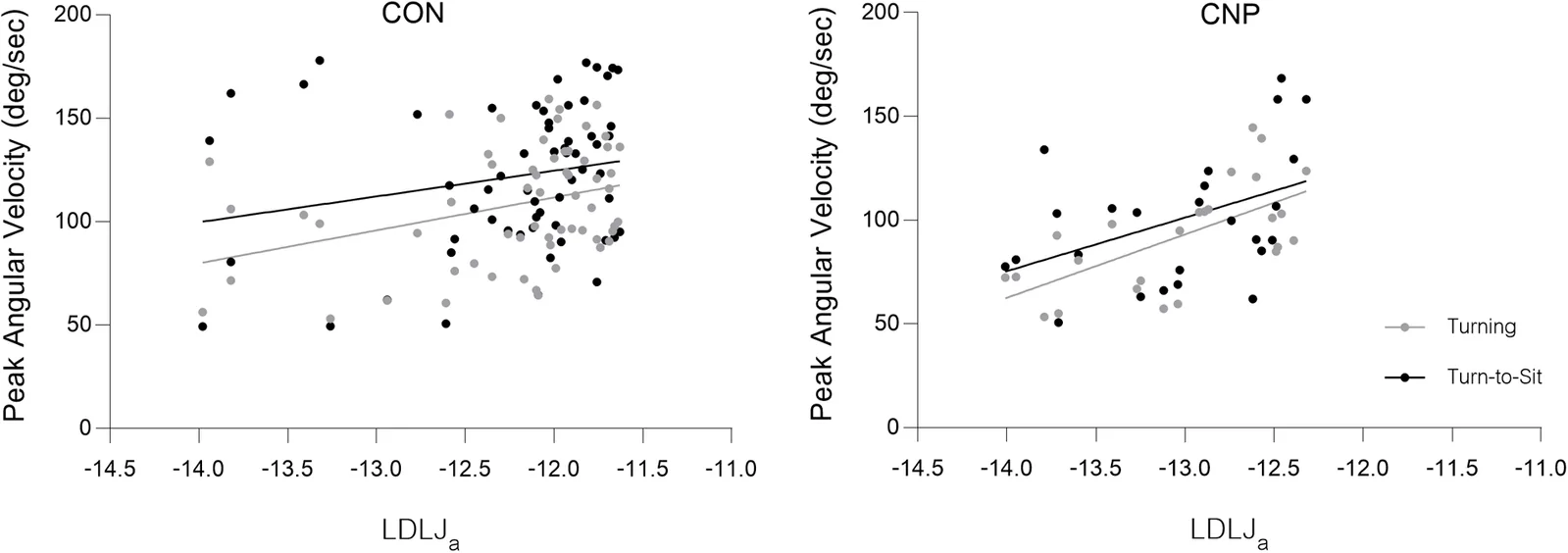
Correlation between peak angular velocity and Log dimensionless jerk. based on Acceleration in Older Adults with and without CNP. Scatter plots illustrating the relationship between movement smoothness (LDLJa) and peak trunk angular velocity during the turning and turn-to-sit phases for the CON (left) and CNP (right) groups. Please note that the solid trend lines represent linear regression and are provided solely for visual illustration purposes. The statistical associations between variables were formally assessed using Spearman's rank correlation coefficients (r_s).

Peak trunk angular velocity was significantly lower in the CNP group during both the turning and turn-to-sit phases (Table 3). LDLJa was positively correlated with peak trunk angular velocity in both groups (Table 4, Figure 3). Moderate positive correlations were observed during turning in both groups, whereas weaker associations were observed during turn-to-sit.

**Table 3:** Comparison of peak angular velocities during turning and turn-to-Sit between groups.

Variable	CON (*n* = 60)	CNP (*n* = 25)	CON-CNP (95% CI)	*p*-value	Effect size
Turning (degrees/sec)	108.10 ± 28.06	92.24 ± 25.57	15.86 (2.90 to 28.81)	0.017*	0.58
Turn-to-Sit (degrees/sec)	121.80 ± 34.92	100.48 ± 31.60	21.33 (5.23 to 37.42)	0.010*	0.63

CON, older adults without chronic neck pain; CNP, older adults with chronic neck pain.

* *p* < 0.05 indicates a statistically significant difference between the CON and CNP groups.

**Table 4:** Spearman rank correlations between LDLJa and peak trunk angular velocity by group.

Group	Phase	r_s	95% CI	*p*-value
CON (*n* = 60)	Turning	0.31	0.06 to 0.53	0.015*
	Turn-to-Sit	0.25	−0.01 to 0.48	0.050
CNP (*n* = 25)	Turning	0.57	0.21 to 0.79	0.003*
	Turn-to-Sit	0.42	0.02 to 0.71	0.036*

CON, older adults without chronic neck pain; CNP, older adults with chronic neck pain; r_s: spearman rank correlation coefficient.

* *p* < 0.05 indicates a statistically significant difference between the CON and CNP groups.

## 4 Discussion

Older adults with chronic neck pain (CNP) did not differ from controls (CON) in gait velocity, stride length, or cadence, yet they took significantly longer to complete the Time Up and Go (TUG) test (Table 2). Conventional spatiotemporal parameters therefore did not explain the slower overall performance, whereas movement smoothness did: the CNP group demonstrated significantly lower the Log Dimensionless Jerk based on acceleration (LDLJa) values, indicating less smooth trunk motion throughout the task (Figure 1). Phase-specific analysis localized these differences to turning, turn-to-sit, and the walking-back phase that follows the turn, while sit-to-stand and walking forward remains comparable between groups (Figure 2). Peak trunk angular velocity was correspondingly lower during turning and turn-to-sit (Table 3), and movement smoothness (LDLJa) correlated positively with turning velocity in both groups (Table 4). To our knowledge, this is the first study to combine the LDLJa with phase-specific instrumented TUG (iTUG) analysis in older adults with CNP.

The two groups were comparable in age, sex, BMI, and comorbidities. As expected, the CNP group reported greater neck-related disability and pain, lower balance confidence, and more dizziness symptoms than controls, in line with previous evidence linking CNP to impaired cervical proprioception, altered sensorimotor integration, and reduced balance confidence (22–24). These findings indicate that, despite demographic similarity, individuals with CNP present clinical features of sensorimotor dysfunction that likely underline the altered movement performance observed in this study.

Although overall gait performance appeared preserved, participants with CNP required significantly longer to complete the TUG, indicating reduced efficiency during complex functional mobility. The preservation of gait velocity, stride length, and cadence suggests that straight-line walking remains relatively intact, whereas dynamic postural transitions impose greater sensorimotor demands. This discrepancy may reflect compensatory strategies, including reduced trunk rotation to minimize cervical loading, increased reliance on visual input to compensate for impaired cervical proprioception (22), and a generally more cautious movement strategy that becomes insufficient when tasks require coordinated head–trunk reorientation, such as turning (3).

This interpretation is consistent with previous studies demonstrating minimal gait impairment during straight walking but more pronounced deficits when additional sensorimotor demands, such as head movement or dual-tasking, are introduced (3). Similarly, Poole et al. and Quek et al. reported that cervical sensorimotor deficits primarily affect dynamic balance rather than linear gait (4, 25). A recent systematic review further concluded that gait impairments in individuals with CNP are most evident during dual-task or head-movement conditions, with consistently large effect sizes (26). Together, these findings suggest that conventional spatiotemporal gait measures may fail to detect mobility deficits that become apparent only during more challenging functional tasks.

The greater sensitivity of LDLJa further supports this interpretation. Participants with CNP exhibited significantly lower LDLJa values, indicating more irregular trunk motion and less coordinated whole-body reorientation. The magnitude of this difference is larger than a logarithmic axis suggests, because the LDLJa is the negative natural logarithm of the dimensionless jerk cost, the Hodges–Lehmann median difference of 0.79 units corresponds to approximately 2.2 times the accumulated normalized jerk in the CNP group over the same task (95% CI, 1.8 to 2.7). For context, differences of 0.27 to 0.30 units distinguish people with multiple sclerosis from matched healthy participants, in whom an improvement of approximately 4% has been proposed as the minimal clinically important difference for this metric (27). Absolute LDLJa values are not comparable across studies, as they depend on the signal analyzed and the length of the analysis window, but the present difference and its effect size are at least comparable to those considered clinically meaningful in that population. These findings agree with previous work showing that jerk-based metrics provide a relatively speed-independent assessment of movement quality (10), and that similar reductions occur in older adults with balance or proprioceptive impairments, where compensatory trunk stiffening and reduced trunk rotation are adopted to enhance perceived stability (28–30). Inertial sensor-derived smoothness indices also show good agreement with laboratory-based assessments and responsiveness to rehabilitation (12–14).

The influence of movement duration on the movement smoothness estimate also warrants consideration, given that participants with CNP required more time to complete the TUG. Traditional jerk-based metrics are highly sensitive to movement duration and amplitude, whereas LDLJa normalizes integrated jerk with respect to both movement duration and peak acceleration, thereby reducing these influences. Nevertheless, this dimensionless formulation minimizes, rather than completely eliminates, the effect of movement duration. Accordingly, the lower LDLJa observed in the CNP group is more likely to reflect an altered motor control strategy; characterized by increased corrective sub-movements, movement hesitations, or trunk stiffening to protect the cervical spine, than simply a mechanical consequence of slower task execution.

Phase-specific analysis further localized these movement impairments. Participants with CNP required significantly longer to complete the turning, walking-back, and turn-to-sit phases, indicating that deficits emerged primarily during tasks requiring dynamic body reorientation rather than during straightforward walking. Turning requires rapid coordination between the head and trunk while maintaining postural stability, making it particularly challenging for individuals with CNP. Pain, stiffness, and impaired cervical proprioception may promote an “en bloc” movement strategy, in which the head and trunk rotate as a single rigid unit to minimize cervical motion (22, 28). Although this strategy may reduce cervical loading, it also limits intersegmental rotation and trunk angular momentum, thereby prolonging turning performance.

An additional finding was the reduced between-participant variability in turning performance. Turning duration was not only longer in the CNP group but also substantially less variable than in controls (SD 0.27 s vs. 0.46 s), with an even greater reduction observed during the subsequent walking-back phase. This narrowing of variability was specific to tasks involving body rotation. In contrast, participants with CNP showed greater variability during sit-to-stand, whereas no between-group differences in variability were observed during walking forward or turn-to-sit. Because the same participants completed every phase of the TUG, these contrasting patterns are unlikely to be explained solely by the smaller sample size of the CNP group. Instead, they suggest that individuals with CNP may converge on a more constrained turning strategy, whereas healthy older adults retain greater flexibility in selecting movement solutions. Under this interpretation, reduced between-participant variability may reflect convergence on a constrained movement strategy, which in turn could indicate reduced flexibility of motor control, consistent with the lower trunk angular velocity and reduced movement smoothness observed during turning.

This interpretation should nevertheless be considered exploratory. Reduced between-participant variability may also reflect pain-related caution, lower habitual physical activity, or a narrower range of functional ability among participants with CNP. Moreover, the present analysis evaluated variability between individuals rather than trial-to-trial variability within individuals, which provides a more direct measure of motor control consistency. Future studies should quantify both forms of variability and examine their relationships with cervical sensorimotor function to better understand the mechanisms underlying these findings.

Although the walking-back phase consists of straight-line walking, its prolongation may represent a carry-over effect of the preceding 180° turn rather than impaired walking itself. Following turning, the sensorimotor system must rapidly re-establish body orientation before forward progression can resume, a process that may be delayed in individuals with CNP because impaired cervical proprioception reduces the accuracy of positional feedback (22–24). Consistent with this interpretation, previous studies of cervical dizziness have shown that rapid head movements increase gait variability and promote more cautious walking compared with undisturbed gait (31). Residual postural instability following turning may therefore contribute to the prolonged walking-back duration observed in the CNP group.

The prolonged turn-to-sit phase warrants particular attention because this subtask comprises a continuous transition in which the final turn and descent into the chair are performed simultaneously rather than as separate movements. Weiss and colleagues demonstrated that older adults adopt two principal strategies during this transition: some complete the turn before initiating sitting, whereas others begin to descend while the turn is still in progress (32). Both excessive separation and prolonged overlap between these components were associated with poorer motor and cognitive performance, and greater movement rigidity was specifically linked to poorer performance of the overlapping strategy (32). This framework provides a plausible explanation for our findings. If individuals with CNP adopt an “en bloc” strategy to minimize cervical motion, coordinating axial rotation with the sagittal-plane descent becomes more difficult, resulting in a more sequential rather than overlapping movement pattern. Such a strategy would prolong the overall turn-to-sit duration without necessarily slowing either component individually, consistent with the reduced peak trunk angular velocity observed during this phase. It may also explain why turn-to-sit, unlike turning, did not exhibit reduced between-participant variability, as both sequential and overlapping strategies remain viable movement solutions. Because Weiss et al. studied a considerably older cohort and focused primarily on cognitive rather than cervical impairment, this interpretation should be regarded as conceptual rather than direct.

The lower peak trunk angular velocities observed during turning and turn-to-sit indicate slower rotational movement and reduced trunk coordination in participants with CNP, consistent with previous evidence that chronic neck pain restricts cervical mobility and alters head–trunk coupling (22, 25, 28). These tasks require rapid body reorientation with controlled trunk rotation and deceleration, processes that rely heavily on cervical proprioceptive and vestibular input (24, 33). Consequently, the reduced angular velocity likely reflects a cautious movement strategy adopted to maintain head–trunk stability during rotation. Clinically, this finding is important because turning angular velocity has been identified as a sensitive indicator of subtle mobility impairment that may not be detected using total TUG time alone (30, 34).

Movement smoothness and turning velocity were closely related in both groups, with higher LDLJa values associated with faster and more coordinated turning. These findings suggest that reduced movement smoothness and slower rotation represent complementary manifestations of the same underlying sensorimotor impairment rather than independent abnormalities. Although the correlation coefficients were numerically larger in the CNP group, the confidence intervals overlapped substantially, and no formal comparison between correlation coefficients was performed. Therefore, we do not infer that the strength of the association differed between groups. Overall, these findings support previous evidence that jerk-based measures of movement smoothness provide a sensitive index of motor control during demanding functional transitions such as turning (11, 12).

From a fall-risk perspective, the median TUG time of the CNP group (12.84 s) exceeded the commonly cited screening threshold of approximately 12 s for community-dwelling older adults, whereas the control group median (11.35 s) remained below this value (35, 36). Together with the observed reductions in movement smoothness and turning velocity, these findings suggest greater mobility impairment during daily activities that require changes in direction or body orientation. Recent instrumented TUG studies have similarly demonstrated that sensor-derived turning metrics improve the identification of mobility deficits beyond total TUG time alone (12, 37). Nevertheless, these findings should be interpreted cautiously. The commonly used TUG cut-off values are intended as screening tools rather than definitive predictors of future falls, and prospective falls were not monitored in the present cross-sectional study. Accordingly, our results indicate impaired mobility with potential relevance to fall risk rather than confirming an increased risk of future falls.

The present findings also have important clinical implications. The phase-specific impairments observed during turning and turn-to-sit indicate that clinicians should not rely solely on total TUG time when evaluating mobility in older adults with CNP. The reduction in movement smoothness itself cannot be judged by observation, since the LDLJa is the time integral of the squared derivative of acceleration; the accompanying prolongation of the turning phase, by contrast, is likely to be noticeable to an experienced clinician. Apparently normal straight-line walking should therefore not be interpreted as evidence of intact sensorimotor control, even in individuals with only mild neck disability, and the LDLJa contributes a graded, objective index of movement quality that observation alone cannot provide. Incorporating phase-specific assessment of dynamic postural transitions into routine clinical evaluation may facilitate earlier identification of mobility deficits and help target rehabilitation toward specific movement impairments. Furthermore, movement smoothness and turning velocity may serve as complementary outcome measures for interventions aimed at improving functional mobility. As accelerometers become increasingly accessible through widespread wearable technologies, such as smartphones and smartwatches, evaluating and calculating smoothness metrics will become seamlessly integrated into routine clinical environments, rather than being confined solely to the laboratory.

Some potential confounders and mediators should also be considered when interpreting these findings. Compared with the control group, participants with CNP reported greater pain intensity (VAS), higher neck-related disability (NDI), lower balance confidence (ABC scale), and more dizziness symptoms (DHI). Rather than representing isolated clinical characteristics, these factors may collectively contribute to impaired dynamic mobility. For example, reduced balance confidence and subclinical dizziness may promote more cautious movement strategies, resulting in prolonged TUG performance and reduced trunk smoothness. Likewise, pain intensity, medication use, and the chronicity or laterality of neck pain may further influence gait performance. In addition, unmeasured psychological factors, such as kinesiophobia, may have contributed to the observed movement alterations. Although these factors are plausible contributors, their relationships with phase-specific TUG performance were not examined in the present study. Future studies with larger samples should investigate these associations to clarify the relative contributions of clinical, sensorimotor, and psychological factors to mobility impairment in older adults with CNP.

This study has several limitations. First, the cross-sectional design precludes causal inference; therefore, longitudinal studies are needed to determine whether chronic neck pain contributes to progressive mobility decline. Second, participants were recruited using convenience sampling, and the groups were unequal in size (CNP, *n* = 25; CON, *n* = 60), which may limit representativeness and reduce the precision of estimates for the CNP group. Furthermore, participants with CNP had only mild neck-related disability (mean NDI = 15.28%); consequently, the findings may not extend to individuals with moderate or severe disability. The predominantly female sample also limits the broader applicability of these findings and highlights the need for more sex-balanced cohorts. Third, the absence of a cervical sensor prevented direct assessment of head–trunk coupling and the proposed “en bloc” movement strategy. In addition, cervical proprioception and vestibular function were not evaluated using dedicated clinical tests; consequently, the sensorimotor mechanisms proposed in this study remain speculative rather than directly demonstrated. Finally, the turn-to-sit phase was analyzed as a single temporal measure. Because turning and sitting involve distinct rotational and translational components, future studies should separate these subphases to better characterize their temporal coordination and identify the specific movement deficits contributing to impaired performance.

## 5 Conclusions

Older adults with chronic neck pain exhibited impaired functional mobility characterized by prolonged TUG performance and reduced movement smoothness, despite preserved basic spatiotemporal gait parameters. These deficits were most pronounced during turning and turn-to-sit, highlighting the importance of dynamic postural transitions in this population. Phase-specific iTUG metrics and LDLJa provide complementary information on movement quality beyond conventional TUG outcomes and may enhance the clinical assessment of mobility impairment in older adults with chronic neck pain.

## Data Availability

The raw data supporting the conclusions of this article will be made available by the authors, without undue reservation.
